# Correction: PaCBAM: fast and scalable processing of whole exome and targeted sequencing data

**DOI:** 10.1186/s12864-024-10348-5

**Published:** 2024-05-13

**Authors:** Samuel Valentini, Tarcisio Fedrizzi, Francesca Demichelis, Alessandro Romanel

**Affiliations:** 1 Laboratory of Bioinformatics and Computational Genomics, Department of Cellular, Computational and Integrative Biology (CIBIO), University of Trento, Trento, Italy; 2 Laboratory of Computational and Functional Oncology, Department of Cellular, Computational and Integrative Biology (CIBIO), University of Trento, Trento, Italy

**Correction** **:** **BMC Genomics 20, 1018 (2019)**

**https://doi.org/10.1186/s12864-019-6386-6**

The wrong Supplementary file was originally published with this article [1]; it has now been replaced with the correct file.

The correct Additional file 1 is also included in this Correction and the original article has been updated.

### Supplementary Information

**Additional file 1:** **Figure S1. **Genomic region mean coverage computation. **Figure S2. **Cumulative coverage distribution report. **Figure S3.**Variant allelic fraction distribution report. **Figure S4.** SNP allelic fraction distribution report. **Figure S5. **Alternative bases distribution report. **Figure S6. **Strand bias distribution report. **Figure S7. **Genomic regions depth of coverage distribution report. **Figure S8. **Genomic regions GC content distribution report. **Figure S9. **Run time comparison at 150X depth of coverage. **Figure S10. **Run time comparison at 230X depth of coverage. **Figure S11. **Run time comparison at 300X depth of coverage. **Figure S12. **Memory usage comparison at 150X depth of coverage. **Figure S13. **Memory usage comparison at 230X depth of coverage. **Figure S14. **Memory usage comparison at 300X depth of coverage. **Figure S15. **Memory usage comparison among PaCBAM pileup and pileup module of ASEQ. **Figure S16. **Comparison of PaCBAM duplicates filtering strategy to Sambamba markdup and Picard MarkDuplicates modules. **Figure S17. **Performance of PaCBAM duplicated reads filtering. **Table S1.** Mean depth of coverage and target sizes of all BAM files used to test PaCBAM performance. **Table S2.** Time and memory usage of duplicates filtering performance analyses. **Table S3.** Versions of the tools used in performance evaluation analysis.
