# Supplementary material for: PaCBAM: fast and scalable processing of whole exome and targeted sequencing data
Source: BMC Genomics. 2019 Dec 26;20:1018. doi: 10.1186/s12864-019-6386-6 (PMC6933905; doi:10.1186/s12864-019-6386-6)
Supplement: Supplementary file 1 — Additional file 1: Figure S1. Genomic region mean coverage computation. Figure S2. Cumulative coverage distribution report. Figure S3. Variant allelic fraction distribution report. Figure S4. SNP allelic fraction distribution report. Figure S5. Alternative bases distribution report. Figure S6. Strand bias distribution report. Figure S7. Genomic regions depth of coverage distribution report. Figure S8. Genomic regions GC content distribution report. Figure S9. Run time comparison at 150X depth of coverage. Figure S10. Run time comparison at 230X depth of coverage. Figure S11. Run time comparison at 300X depth of coverage. Figure S12. Memory usage comparison at 150X depth of coverage. Figure S13. Memory usage comparison at 230X depth of coverage. Figure S14. Memory usage comparison at 300X depth of coverage. Figure S15. Memory usage comparison among PaCBAM pileup and pileup module of ASEQ. Figure S16. Comparison of PaCBAM duplicates filtering strategy to Sambamba markdup and Picard MarkDuplicates modules. Figure S17. Performance of PaCBAM duplicated reads filtering. Table S1. Mean depth of coverage and target sizes of all BAM files used to test PaCBAM performance.Table S2. Time and memory usage of duplicates filtering performance analyses. Table S3. Versions of the tools used in performance evaluation analysis. [file 12864_2019_6386_MOESM1_ESM.pdf]

DOI: 10.1186/s12864-019-6386-6

File Correction Details

Correction is made. No of Corrections: 71

Online Correction Link : **https://eproofing.springer.com/journals_v2/index.php?token=3r6TkXtPts7Zal6LvJYqOa3fBaeoWO4vYal2Dmn-Z8hUbt6W6JgEoQ**

Image Annotation Details

No Details Found

Attached File Details

Figure1.pdf Supplementary_Material_16122019.pdf

Query Details

1. Author names: Please confirm if the author names are presented accurately (given names/initials, family name). Author 1: Given name: Samuel Family name: Valentini Author 2: Given name: Tarcisio Family name: Fedrizzi Author 3: Given name: Francesca Family name: Demichelis Author 4: Given name: Alessandro Family name: Romanel

OK

2. As per standard instruction, city and country is required for affiliations; however, this information is missing in affiliation 1 and 2. Please check if the provided city and country is correct and amend if necessary.

Correct

3. Figures 1 and 2 contains small text. Please provide replacement figure file. Otherwise, kindly confirmed if we can retain the current presentation.

A new Figure1 has been uploaded. Figure2 is ok as it is.

4. Supplementary Figure S1-S17 and Table S1-S3 citations were changed to Additional file1: Figure S1-S17 and Table S1-S3 to avoid confusion with any figures and tables included within the main text. Moreover, titles inside the additional files were also amended to correspond with their modified citations. Please check if appropriate.

Appropriate

5. As per journal requirements, every additional file must have a corresponding caption. In this regard, please be informed that the caption was taken from the additional e-file itself. Please advise if the action taken is appropriate and amend if necessary.

Captions of figures in Additional file 1 have been updated. An updated Additional file 1 "Supplementary_Materials_16122019.pdf" has been uploaded.

6. Journal standard requires Additional file legends of less than 300 words only; however, the provided data in the manuscript exceed the requirement. Please shorten the additional file legend accordingly. We suggest that any detailed information should be included as an introduction within the Additional file itself.

Captions of figures in Additional file 1 have been updated and Additional file legends in the main text have been corrected accordingly. They should now respect the limit of 300 words.

10.1186/s12864-019-6386-6

PaCBAM: fast and scalable processing of whole exome and targeted sequencing data

Open Access This article is distributed under the terms of the Creative Commons Attribution 4.0 International License (http://creativecommons.org/licenses/by/4.0/), which permits unrestricted use, distribution, and reproduction in any medium, provided you give appropriate credit to the original author(s) and the source, provide a link to the Creative Commons license, and indicate if changes were made. The Creative Commons Public Domain Dedication waiver (http://creativecommons.org/publicdomain/zero/1.0/) applies to the data made available in this article, unless otherwise stated.

Samuel Valentini, [1]

Email samuel.valentini@unitn.it

AQ1

Tarcisio Fedrizzi, [2]

Email tarcisio.fedrizzi@unitn.it

Francesca Demichelis, [2]

Email f.demichelis@unitn.it

Alessandro Romanel, [1]✉

Email alessandro.romanel@unitn.it

[1] Laboratory of Bioinformatics and Computational Genomics, Department of Cellular, Computational and Integrative Biology (CIBIO) , University of Trento, Trento, Italy

[2] Laboratory of Computational and Functional Oncology, Department of Cellular, Computational and Integrative Biology (CIBIO) , University of Trento, Trento, Italy **AQ2**

Received: 27 March 2019 / Accepted: 11 December 2019

Abstract

Background

Interrogation of whole exome and targeted sequencing NGS data is rapidly becoming a preferred approach for the exploration of large cohorts in the research setting and importantly in the context of precision medicine. Single-base and genomic region level data retrieval and processing still constitute major bottlenecks in NGS data analysis. Fast and scalable tools are hence needed.

Results

PaCBAM is a command line tool written in C and designed for the characterization of genomic regions and single nucleotide positions from whole exome and targeted sequencing data. PaCBAM computes depth of coverage and allele-specific pileup statistics, implements a fast and scalable multi-core computational engine, introduces an innovative and efficient *on-the-fly* read duplicates filtering strategy and provides comprehensive text output files and visual reports. We demonstrate that PaCBAM exploits parallel computation resources better than existing tools, resulting in important reductions of processing time and memory usage, hence enabling an efficient and fast exploration of large datasets.

Conclusions

PaCBAM is a fast and scalable tool designed to process genomic regions from NGS data files and generate coverage and pileup comprehensive statistics for downstream analysis. The tool can be easily integrated in NGS processing pipelines and is available from Bitbucket and Docker/Singularity hubs~~ under the MIT license~~.

Abbreviations

NGS	Next-Generation Sequencing
SNP	Single Nucleotide ~~p~~Polymorphism
VAF	Variant(s) Allele Frequency
WES	Whole-Exome Sequencing

Background

Genomic region and single-base level data retrieval and processing, which represent fundamental steps in genomic analyses such as copy number estimation, variant calling and quality control, still constitute one of the major bottlenecks in NGS data analysis. To deal with the computationally intensive task of calculating depth of coverage and pileup statistics at specific chromosomal regions and/or positions, different tools have been developed. Most of them, including specific modules of SAM~~T~~tools [1] and BEDTools [2] and the most recent Mosdepth [3], only measure and optimize the computation of depth of sequencing coverage. Few others, like the *pileup* modules of SAM~~T~~tools, Sambamba [4], GATK [5] and ASEQ [6] provide instead statistics at single-base resolution, which is essential to perform variant calling, allele-specific analyses and exhaustive quality control. Although most of these tools offer parallel computation options, scalability in terms of memory and multiple processes/threads usage is still limited. To enable an efficient exploration of large scale NGS datasets, here we introduce PaCBAM, a tool that provides fast and scalable processing of targeted re-sequencing data of varying sizes, from WES to small gene panels. Specifically, PaCBAM computes depth of coverage and allele-specific pileup statistics at regions and single-base resolution levels and provides data summary visual reporting utilities. PaCBAM introduces also an innovative and efficient *on-the-fly* read duplicates filtering approach. While most tools for read duplicates filtering work on SAM/BAM files sorted by read name [1, 7] or read position (Tarasov et al., 2015, broadinstitute.github.io~~-~~/picard) and generate new SAM/BAM files, PACBAM performs the filtering directly during the processing, not requiring the creation of intermediate BAM/SAM files and fully exploiting parallel resources.

Implementation

PaCBAM is a command line tool written in C programming language that combines multi-threaded computation, SAMTools APIs, and an ad-hoc data structures implementation. PaCBAM expects as input a sorted and indexed BAM file, a sorted BED file with the coordinates of genomic regions (namely the *target*, e.g. captured regions of a WES experiment), a VCF file specifying a list of SNPs of interest within the *target* and a reference genome in FASTA format. PaCBAM implements a multi-threaded solution that optimizes the execution time when multiple cores are available. The tool splits the list of regions provided in the BED file and spawns different threads to execute parallel computations using a shared and optimized data structure. The shared data structure collects both region and single-base level information and statistics which are processed and finally exposed through four different output options. Each output mode provides the user with only the statistics of

interest, generating a combination of the following text output files: a) *depth of coverage of all genomic regions* , which for each region provides the mean depth of coverage, the GC content and the mean depth of coverage of the sub-region (user specified, default 0.5 fraction) that maximizes the coverage peak signal, to account for the reduced coverage depth due to incomplete match of reads to the captured regions (Additional file 1: Figure S1); b) *single-base resolution pileup*, which provides for each genomic position in the target the read depth for the 4 possible bases (A, C, G and T), the total depth of coverage, the variants allelic fraction (VAF), the strand bias information for each base; c) *pileup of positions with alternative base support* , which extracts the pileup statistics only for positions with positive VAF, computed using the alternative base with highest coverage (if any); d) *pileup of SNPs positions* , which extracts the pileup statistics for all SNPs specified in the input VCF file and uses the alternative alleles specified in the VCF file for the VAF calculation and the genotype assignment (Additional file 1 for details). All output files are tab-delimited text files and their format details are provided in the Additional file 1.

PaCBAM allows the user to specify the minimum base quality score and the minimum read mapping quality to filter out reads during the pileup processing.

In addition, we implemented an efficient *on-the-fly* duplicated reads filtering strategy which implements an approach that is similar to the Picard MarkDuplicates method but that applies the filter during region and single-base level information retrieval and processing without the need of creating new BAM files (Additional file 1). The filtering strategy, which fully exploits multi-core capabilities, uses single or paired read alignment positions (corrected for soft-clipping at the 5′ end) and total mapping size information to identify duplicates and implements ad-hoc data structures to obtain computational efficiency.

PaCBAM package also includes a Python script to generate visual data reports which can be directly used for quality control. Reports include plots summarizing distributions of regions and per-base depth of coverage, SNPs VAF distribution and genotyping, strand bias distribution, substitutions spectra, regions GC content (Additional file 1: Figure S~~1~~2-S8).

Results

PaCBAM performances were tested on an AMD Opteron 6380 32-cores machine with 256 GB RAM. To mimic different application scenarios, we measured the execution time and memory used by PaCBAM to compute pileups from multiple input BAM files spanning different depth of coverage and different target sizes (Additional file 1: Table S1) using an increasing number of threads. We compared PACBAM performances against pileup modules of SAM~~T~~tools, Sambamba and GATK (SAM~~T~~tools offer no parallel pileup option).

In terms of runtime, as shown in Fig. 1a and Additional file 1: Figure S9-S11, PaCBAM and Sambamba are the only tools that scale with the number of threads used. PaCBAM outperforms all other tools in all tested conditions. Of note, while PaCBAM pileup output files are of constant size, output files of SAM ~~T~~tools, Sambamba and GATK have a size that is function of the coverage; among all the experiments we run in the performance analyses, PaCBAM output is up to 17.5x smaller with respect to outputs generated by the other tested tools.

Fig. 1

PaCBAM performances. Time (**a**) and memory (**b**) required by PaCBAM to perform a pileup compared to SAM ~~T~~tools, GATK and Sambamba, using increasing number of threads. The figure focuses on the analysis of a BAM file with ~300~~x~~X mean coverage and ~30Mbp target size using 30 threads. Note that parallel pileup option is not available for SAM~~T~~tools and red lines in panel **a** and **b** refer to the average of single thread executions

AQ3

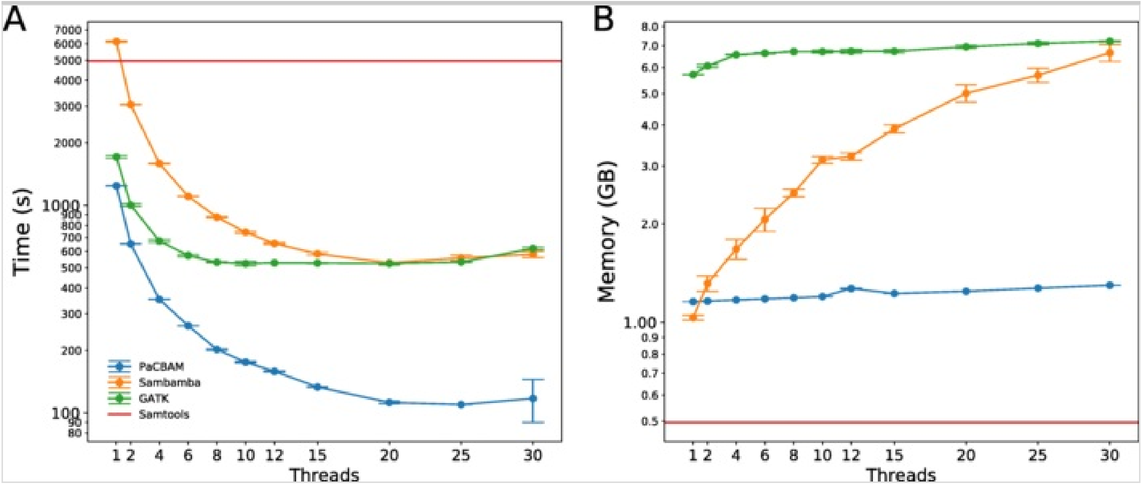

While GATK and PaCBAM, as shown in Fig. 1b and Additional file 1: Figure S12-S14, have a memory usage that depends only on the target size, Sambamba usage depends on both target size and number of threads and SAM~~T~~tools usage is constant. Above 8 cores, PaCBAM beats both GATK and Sambamba in all tested conditions in memory usage.

As an example of performance comparison, when analyzing a BAM file with ~300X mean coverage and ~30Mbp target size using 30 threads (Fig. 1a-b), PaCBAM improves execution time of 4.9x/5.27x and requires 80%/82% less memory compared to Sambamba/GATK.

Of note, in the sequencing scenarios here considered, PaCBAM demonstrates up to 100x execution time improvement and up to 90% less memory usage with respect to the single-base pileup module of our previous tool ASEQ (Additional file 1: Figure S15).

Depth of coverage and pileup statistics of PaCBAM pileup were compared to GATK results on a BAM file with ~300X average coverage and ~64Mbp target size observing almost perfect concordance (Fig. 2a-b).

Fig. 2

Comparison of PaCBAM results with other tools. **a** Comparison of PaCBAM and GATK depth of coverage (left) with zoom in the coverage range [0,500] (right); number of positions considered in the analysis and correlation results are reported. **b** Comparison of allelic fraction of ~ 40 K positions annotated as SNPs in dbSNP database v144 and having an allelic fraction > 0.2 in both PaCBAM and GATK pileup output. **c** Single-base coverage obtained by running either *Picard MarkDuplicates + PaCBAM pileup* or *PaCBAM pileup with duplicates filtering* option active (left) with zoom in the coverage range [0,500] (right). **d** Regional mean depth of coverage obtained by running either *Picard MarkDuplicates + PaCBAM pileup* or *PaCBAM pileup with duplicates filtering* option active

10.1186/s12864-019-6386-6

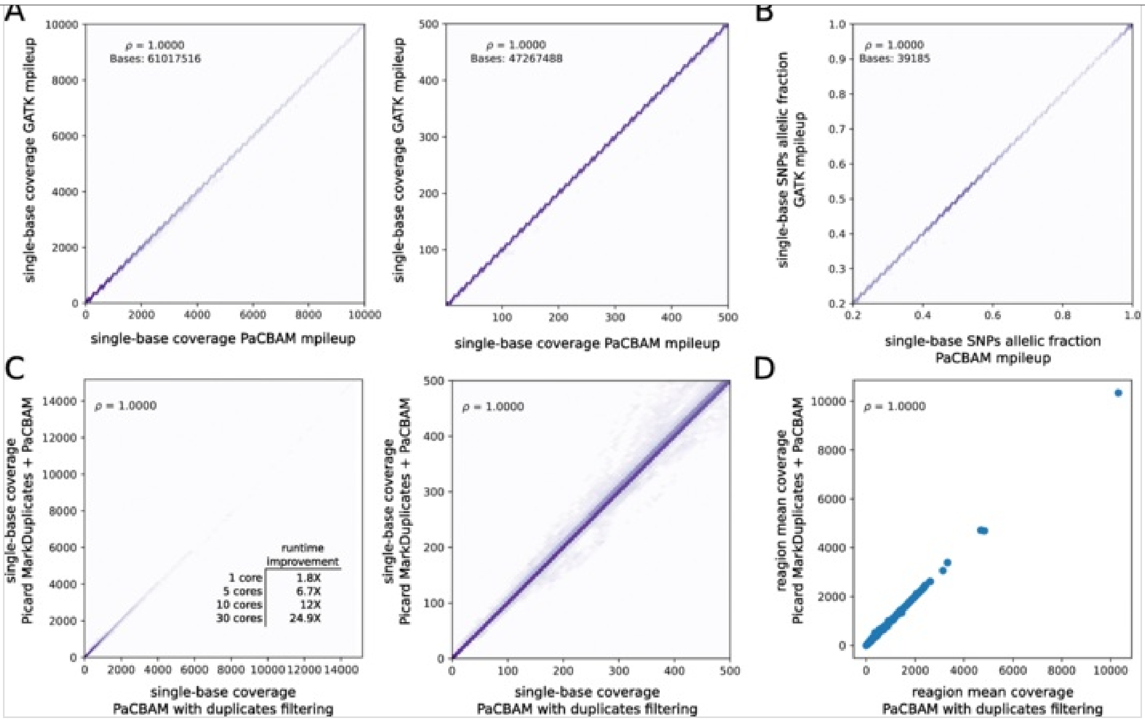

PaCBAM duplicates removal strategy was tested by comparing PaCBAM pileups obtained from a paired-end BAM file first processed with Picard MarkDuplicates or parallel Sambamba markdup, to PaCBAM pileups obtained from the same initial BAM file but using the embedded on-the-fly duplicates filtering. As shown in Fig. 2c-d and Additional file 1: Figure S16, both single-base and region level statistics results are strongly concordant, with single-base total coverage difference (with respect to Picard) that in 99.94% of positions is < 10X, single-base allelic fraction difference that in 99.95% of positions is < 1% and region mean coverage difference that in 99.96% of regions is <10X (Additional file 1: Figure S17~~A-B-C~~). In addition, PaCBAM strategy improves overall execution time of 2.5x/1.7x with a single thread and of 25x/3x with 30 threads compared to Picard and parallel Sambamba, respectively (Additional file 1: Table S2, Fig. 2c, Additional file 1: Figure S16A).

Overall, these analyses demonstrate that PaCBAM exploits parallel computation resources better than existing tools, resulting in evident reductions of processing time and memory usage, that enable a fast and efficient coverage and allele-specific characterization of large WES and targeted sequencing datasets. The performance analysis is completely reproducible using an ad-hoc Debian-based Singularity container (Additional file 1: Table S3, Additional file 1).

Conclusion

We presented PaCBAM, a fast and scalable tool to process genomic regions from NGS data files and generate coverage and pileup statistics for downstream analysis such as copy number estimation, variant calling and data quality control. Although designed for targeted re-sequencing data, PaCBAM can be used to characterize any set of genomic regions of interest from NGS data. PaCBAM generates both region and single-base level statistics and provides a fast and innovative *on-the-fly* read duplicates filtering strategy. The tool is easy to use, can be integrated in any NGS pipeline and is available in source/binary version on Bitbucket and containerized from Docker and Singularity hubs.

Availability and requirements

Project name: PaCBAM

Project home page: bcglab.cibio.unitn.it~~-~~/PaCBAM

Operating system(s): Platform independent

Programming language: C, Python

License: MIT

Supplementary information

Supplementary information accompanies this paper at https://doi.org/10.1186/s12864-019-6386-6 .

Publisher's Note

Springer Nature remains neutral with regard to jurisdictional claims in published maps and institutional affiliations.

Acknowledgments

Not applicable.

Authors' contributions

AR designed and implemented PaCBAM. SV designed and implemented visual reporting scripts and performed all performance analyses. TF and FD contributed with tool testing, access to computational resources and performance analyses. AR supervised the project. All authors contributed to the writing and editing of the manuscript and approved the manuscript.

Funding

~~This work has been supported by AIRC MFAG 20621 (A.R.) for the design, implementation and performance analysis and by the NCI P50 CA211024-01 Weill Cornell Medicine Prostate Cancer SPORE (F.D.) for testing and performance analysis.~~The research leading to these results has received funding from AIRC under MFAG 2017 - ID. 20621 project - P.I. Romanel Alessandro - for the design, implementation and performance analyses and from NCI P50 CA211024-01 Weill Cornell Medicine Prostate Cancer SPORE - Demichelis Francesca - for testing and performance analyses.

Availability of data and materials

All data and analysis scripts supporting the results of this article are available at bcglab.cibio.unitn.it/PaCBAM_Performance_Analysis~~from the corresponding author on reasonable request~~.

Ethics approval and consent to participate Not applicable.

Consent for publication Not applicable.

Competing interests The authors declare that they have no competing interests.

Additional file

AQ4
AQ5

Additional file 1: Figure S1.

AQ6

~~Example of region mean coverage computation using a user specified region fraction equal to 0.5 .~~Genomic region mean coverage computation. **Figure S2.** ~~Example of visual report of the cumulative coverage distribution for all positions reported in the PaCBAM pileup output file~~Cumulative coverage distribution report. **Figure S3.** ~~Example of visual report of variants allelic fraction distribution for all positions in the PaCBAM output pileup~~ Variant allelic fraction distribution report. **Figure S4.** ~~Example of visual report of the allelic fraction (AF) distribution of all positions contained in the PaCBAM *.snps output file~~ SNP allelic fraction distribution report. **Figure S5.** ~~Example of visual report of the distribution of alternative bases found for each reference base across all positions reported in the *.pabs PaCBAM output file (i.e. all positions with non-zero variant allelic fraction)~~Alternative bases distribution report. **Figure S6.** ~~Example of PaCBAM visual report of the distribution of strand bias computed across all positions reported in the *.snvs PaCBAM output file (i.e. all positions with non-zero variant allelic fraction)~~Strand bias distribution report. **Figure S7.** ~~Example of visual report of the mean depth of coverage distribution computed across all regions reported in the PaCBAM output file~~ Genomic regions depth of coverage distribution report. **Figure S8.** ~~Example of visual report of the distribution of GC content computed across all regions reported in the *.rc PaCBAM output file~~Genomic regions GC content distribution report. **Figure S9.** ~~Run time comparison among PaCBAM pileup and pileup module of SAMTools, GATK and Sambamba~~Run time comparison at 150X depth of coverage. **Figure S10.** ~~Run time comparison among PaCBAM pileup and pileup module of SAMTools, GATK and Sambamba~~Run time comparison at 230X depth of coverage. **Figure S11.** ~~Run time comparison among PaCBAM pileup and pileup module of SAMTools, GATK and Sambamba.~~ Run time comparison at 300X depth of coverage. **Figure S12.** ~~Memory usage comparison among PaCBAM pileup and pileup module of SAMTools, GATK and Sambamba~~Memory usage comparison at 150X depth of coverage. **Figure S13.** ~~Memory usage comparison among PaCBAM pileup and pileup module of SAMTools, GATK and Sambamba~~ Memory usage comparison at 230X depth of coverage. **Figure S14.** ~~Memory usage comparison among PaCBAM pileup and pileup module of SAMTools, GATK and Sambamba .~~Memory usage comparison at 300X depth of coverage. **Figure S15.** Memory usage comparison among PaCBAM pileup and pileup module of ASEQ. **Figure S16.** Comparison of PaCBAM duplicates filtering strategy to ~~s~~Sambamba markdup and Picard MarkDuplicates modules. **Figure S17.** Performance of PaCBAM duplicated reads filtering. **Table S1.** Mean depth of coverage and target sizes of all BAM files used to test PaCBAM performance. **Table S2.** Time and memory usage of ~~PaCBAM, Sambamba and Picard MarkDuplicates for~~ duplicates filtering performance analyses~~and pileup of a paired-end WES experiment~~. **Table S3.** Versions of the tools used in performance evaluation analysis.

References

1. Li H, Handsaker B, Wysoker A, Fennell T, Ruan J, Homer N, et al. The sequence alignment/map format and SAMtools. Bioinformatics. 2009;25:2078–9.

2. Quinlan AR, Hall IM. BEDTools: a flexible suite of utilities for comparing genomic features. Bioinformatics. 2010;26:841–2.

3. Pedersen BS, Quinlan AR. Mosdepth: quick coverage calculation for genomes and exomes. Bioinformatics. 2018;34:867–8.

4. Tarasov A, Vilella AJ, Cuppen E, Nijman IJ, Prins P. Sambamba: fast processing of NGS alignment formats. Bioinformatics. 2015;31:2032–4.

5. DePristo MA, Banks E, Poplin R, Garimella KV, Maguire JR, Hartl C, et al. A framework for variation discovery and genotyping using next-generation DNA sequencing data. Nat Genet. 2011;43:491–8.

6. Romanel A, Lago S, Prandi D, Sboner A, Demichelis F. ASEQ: fast allele-specific studies from next-generation sequencing data. BMC Med Genet. 2015;8. https://doi.org/10.1186/s12920-015-0084-2 .

7. Faust GG, Hall IM. SAMBLASTER: fast duplicate marking and structural variant read extraction. Bioinformatics. 2014;30:2503–5.
