# Supplementary material for: Correction: PaCBAM: fast and scalable processing of whole exome and targeted sequencing data
Source: BMC Genomics. 2024 May 13;25:463. doi: 10.1186/s12864-024-10348-5 (PMC11089790; doi:10.1186/s12864-024-10348-5)
Supplement: Supplementary file 1 — Additional file 1: Figure S1. Genomic region mean coverage computation. Figure S2. Cumulative coverage distribution report. Figure S3.Variant allelic fraction distribution report. Figure S4. SNP allelic fraction distribution report. Figure S5. Alternative bases distribution report. Figure S6. Strand bias distribution report. Figure S7. Genomic regions depth of coverage distribution report. Figure S8. Genomic regions GC content distribution report. Figure S9. Run time comparison at 150X depth of coverage. Figure S10. Run time comparison at 230X depth of coverage. Figure S11. Run time comparison at 300X depth of coverage. Figure S12. Memory usage comparison at 150X depth of coverage. Figure S13. Memory usage comparison at 230X depth of coverage. Figure S14. Memory usage comparison at 300X depth of coverage. Figure S15. Memory usage comparison among PaCBAM pileup and pileup module of ASEQ. Figure S16. Comparison of PaCBAM duplicates filtering strategy to Sambamba markdup and Picard MarkDuplicates modules. Figure S17. Performance of PaCBAM duplicated reads filtering. Table S1. Mean depth of coverage and target sizes of all BAM files used to test PaCBAM performance. Table S2. Time and memory usage of duplicates filtering performance analyses. Table S3. Versions of the tools used in performance evaluation analysis. [file 12864_2024_10348_MOESM1_ESM.pdf]

# PaCBAM: fast and scalable processing of whole exome and targeted sequencing data

Supplementary Material

## 1. Assignment of genotypes to input SNPs

PaCBAM provides an option to assign genotype calls to all SNPs listed in the input VCF file. The tool implements two approaches. The first one is based on the VAF value and specifically assigns genotype 0/0 when VAF<=0.2, assigns genotype 0/1 when VAF is in the range (0.2,0.8) and assigns genotype 1/1 when VAF is >=0.8. The second approach instead implements a binomial test with probabilities p and q for the reference and the alternative allele, respectively, and a significance cutoff at 1%. To account for the reference bias mapping, we apply default probabilities $p = 0.55$ and $q = 0.45$. High precision and recall of these two approaches in genotyping common SNPs, compared to SNP array genotype calls, was previously shown in (Romanel *et al.*, 2015).

## 2. *On-the-fly* read duplicates filtering

PaCBAM duplicates filtering strategy is applied while computing single base and region level statistics and fully exploits parallel computation. To allow duplicated reads identification at a specific captured region *R* with genomic coordinates *chr:start-end*, a preliminary fetching of reads is performed. Specifically, reads in the extended region *chr:(start-W)-(end+W)* are retrieved (with *W* tunable by the user with default value equal to 1,000) and a hash-map collecting positional and mapping information on paired (or single) end reads is populated using the read name as key string. For each read, using the CIGAR value, the alignment position is corrected for soft-clipping at the 5' end and the size of mapped region is calculated. Duplicates filtering is then performed by searching for paired (or single) end reads with same corrected positions and selecting the one with largest total mapped region size as representative for each duplicated reads group. Only selected reads are kept in the hash-map and considered during the second and standard fetching of reads that is used to compute single base and region level statistics. As shown in **Figure S17**, default *W* parameter value equal to 1,000 represents a good trade-off balance between computational performances and duplicates filtering effectiveness when paired-end reads are used. When single-end alignment files are processed *W* can be set to 0.

## 3. Creation of BAM files for performance experiments

PaCBAM has been tested on BAM files representing different target sizes. BAM files were created using different BED files representing the different target sizes. Starting from the original Nimblegen SeqCap EZ Exome v3 kit BED file of size 64,190,747bp containing genomic coordinates of exonic captured regions, new BED files of

sizes 6,424,707bp, 16,053,802bp, 32,102,630bp, and 48,144,328bp (corresponding to 10%, 25%, 50% and 75% of the original BED file, respectively) have been generated using a gene-level random sampling strategy. Random sampling was performed by first annotating the original Nimblegen BED file adding the HUGO symbols of the genes corresponding to the captured regions. Random genes were then uniformly sampled from the set of all genes (without replacement) and all overlapping captured regions were incrementally added to a BED file until the desired target size was reached.

BAM files corresponding to all combinations reported in **Table S2** were created from the 1,000 Genomes Project HG02057 individual FASTQ files. Alignment was performed with BWA (Li and Durbin, 2009), SAMtools were used to create BAM files, GATK was used to perform realignment and recalibration and SAMtools were finally used to fix MD tags. BAM file subsampling was done using SAMtools view command, specifying the fraction of the reads (using -s option) to obtain the desired mean depth of coverage and providing pre-designed BED files (using -L option) to get the desired target representation.

## 4. Design of performance experiments and reproducibility

To allow reproducibility of our performance analysis, we created a Debian-based Singularity container. The container provides a standardized and configured environment with all the dependencies required to run all tools and replicate the overall analysis. All used BAM, BED and VCF files and all implemented scripts are available in the container. The analysis pipeline runs each of tested tools three times for each combination of input parameters. For each run the elapsed real time (wall clock) and the peak memory usage of the process was measured using the GNU time command. No other user process was running on the test machine while collecting performance data. The pipeline also produces automatically all tables and images related with performance evaluation here reported.

## 5. Output files specification

### *2.1 File *.pileup*

For each genomic position specified in the input BED file it provides:

- Contig (e.g. chromosome)
- Genomic coordinate of the position
- Read depth of the 4 possible bases A, C, G and T
- Variant allelic fraction (VAF, considering all alternative bases)
- Total depth of coverage
- Strand bias information for each base (when run option “strandbias” is used)

### *2.2 File *.snps*

For each genomic position specified in the input VCF file and present in the regions specified in the input BED file it provides:

- Contig (e.g. chromosome)
- Genomic coordinate of the position

- Position ID (e.g. rsID) specified in the VCF input file
- Reference and alternative bases
- Read depth of the 4 possible bases A, C, G and T
- Variant allelic fraction (VAF, computed with alternative base specified in the VCF file)
- Total depth of coverage
- Genotype (when run option “genotype” or “genotypeBT” is used)

*2.3 File *.rc*

- Contig (e.g. chromosome)
- Genomic start/end coordinates of the region
- Genomic start/end coordinates of the *peaked* region
- Mean read depth of the region
- Mean read depth of the *peaked* region
- GC content in the region

*2.4 File *.pabs*

- Contig (e.g. chromosome)
- Genomic coordinate of the position
- Reference and alternative bases
- Read depth of the 4 possible bases A, C, G and T
- Variant allelic fraction (VAF, considering all alternative bases)
- Total depth of coverage
- Strand bias information for each base (when run option “strandbias” is used)

# Supplementary Figures

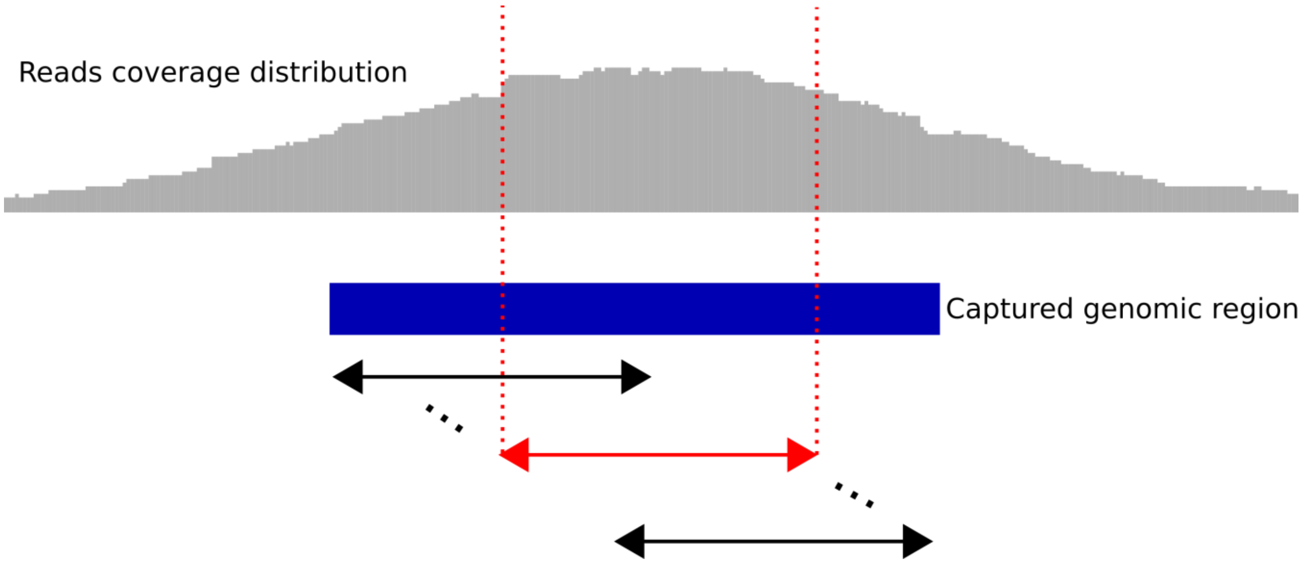


**Figure S1**: Genomic region mean coverage computation. Example of region mean coverage computation using a user specified region fraction equal to 0.5. In red the 0.5 fraction of the region supporting the maximum mean depth of coverage for that region. This value (along with corresponding genomic coordinates) is reported in the PaCBAM output together with the overall region mean depth of coverage.

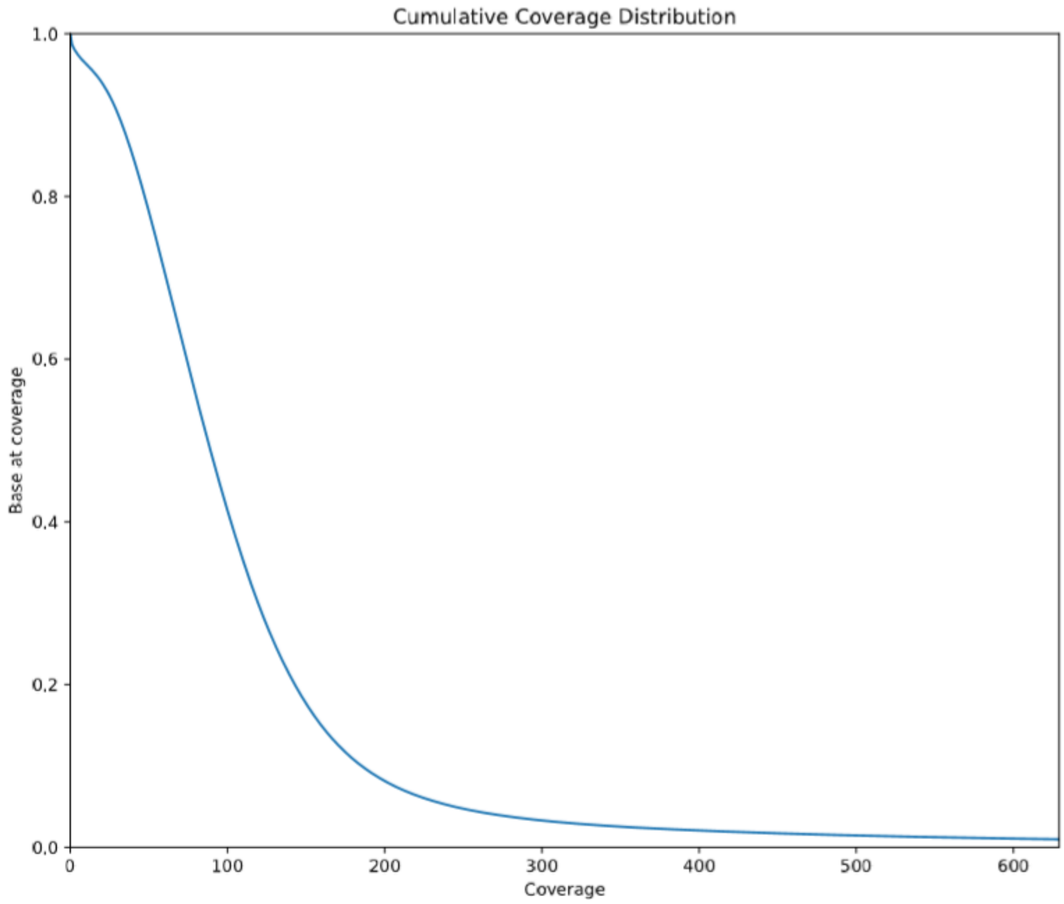


**Figure S2**: Cumulative coverage distribution report. Example of visual report of the cumulative coverage distribution for all positions reported in the PaCBAM pileup output file.

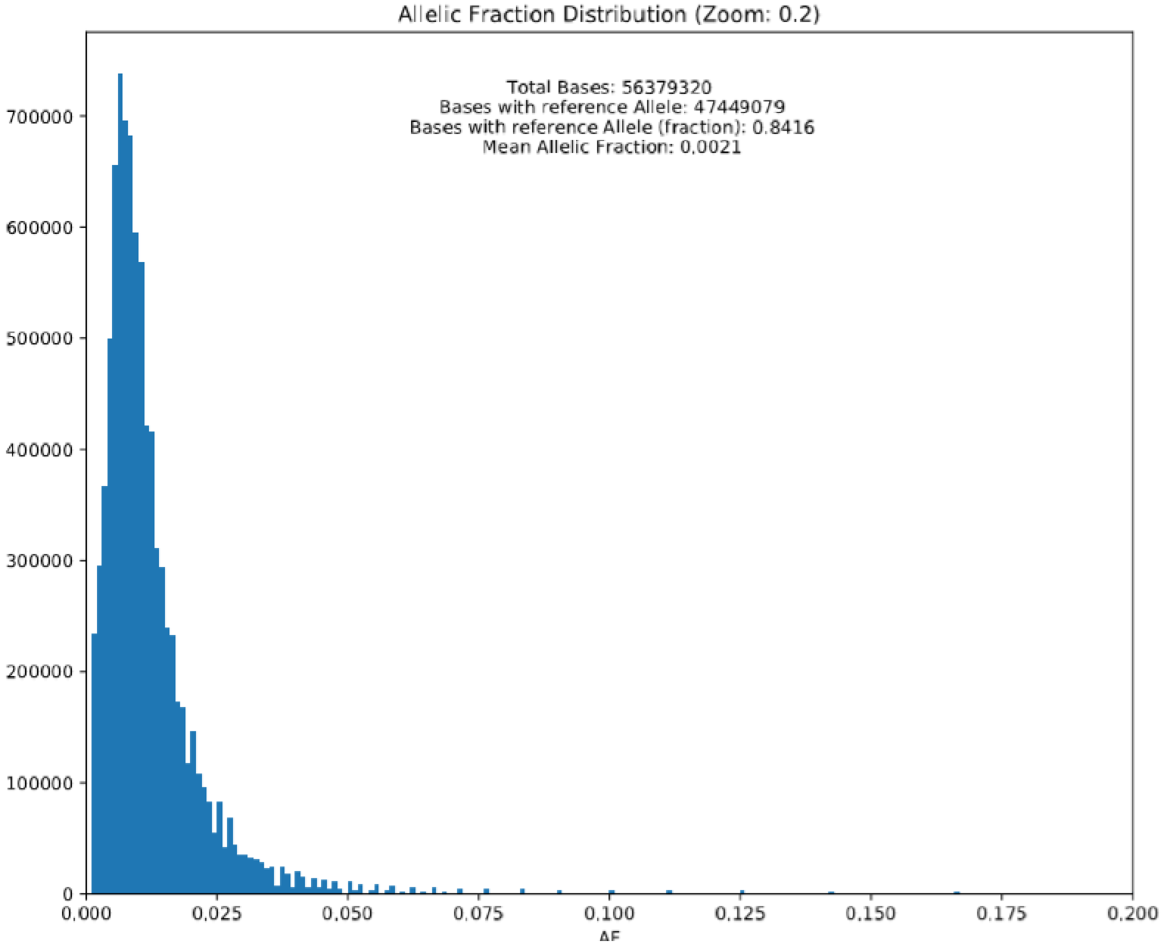


**Figure S3:** Variant allelic fraction distribution report. Example of visual report of variants allelic fraction distribution for all positions in the PaCBAM output pileup. The image shows a detail in the variants allelic fraction range (0-0.2]. This data could provide information regarding the sequencing error distribution for the specific experiment.

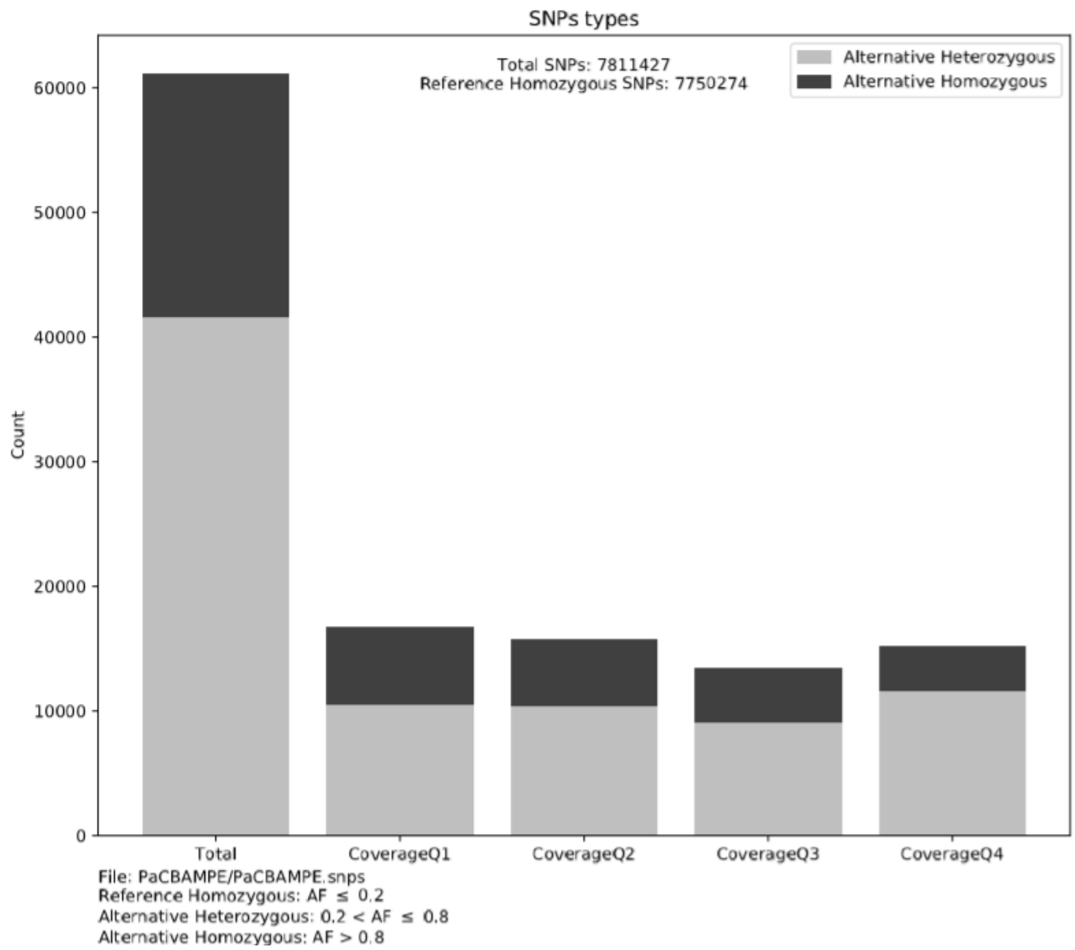


**Figure S4:** SNP allelic fraction distribution report. Example of visual report of the allelic fraction (AF) distribution of all positions contained in the PaCBAM *.snps output file. SNPs are classified as heterozygous or alternative homozygous based on standard AF thresholds. Classification is also reported stratified by coverage quartiles.

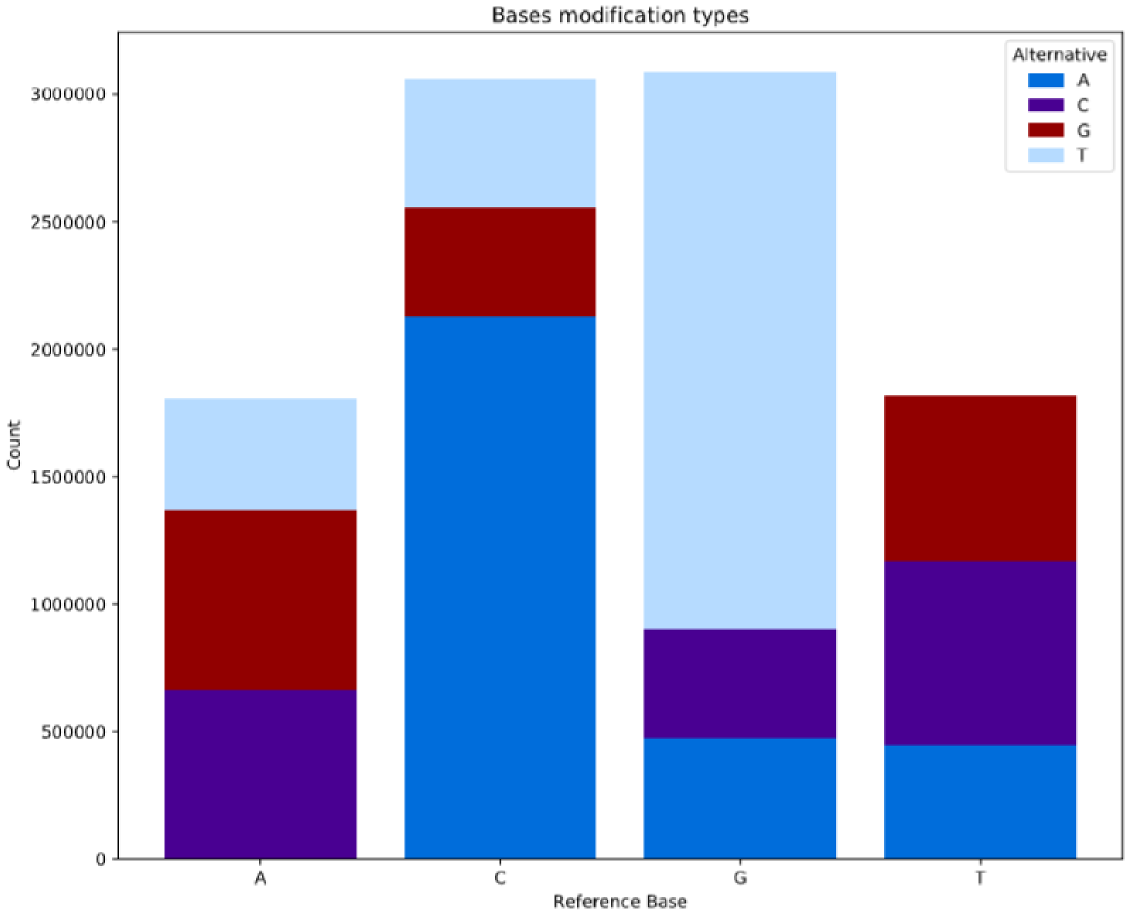


**Figure S5:** Alternative bases distribution report. Example of visual report of the distribution of alternative bases found for each reference base across all positions reported in the *.pabs PaCBAM output file (i.e. all positions with non-zero variant allelic fraction).

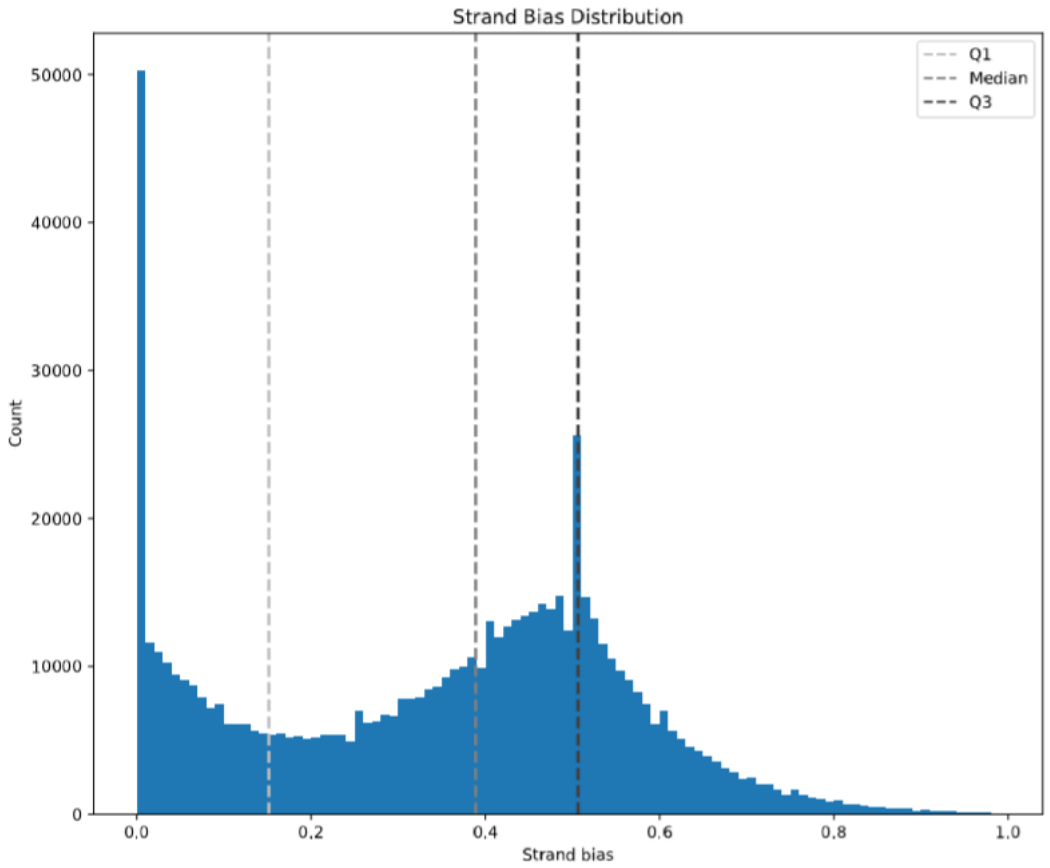


**Figure S6:** Strand bias distribution report. Example of PaCBAM visual report of the distribution of strand bias computed across all positions reported in the *.snvs PaCBAM output file (i.e. all positions with non-zero variant allelic fraction). Strand bias is computed at each position using the formula:

$$abs\left(\frac{REFERENCE_{reverse}}{REFERENCE_{tot}} - \frac{ALTERNATIVE_{reverse}}{ALTERNATIVE_{tot}}\right)$$

which computes the absolute difference between ratio of the number of reverse reads supporting the reference base over the total number of reads supporting the reference base and the ratio of the number of reverse reads supporting the alternative base over the total number of reads supporting the alternative base. Values towards 1 represent strong strand bias for the corresponding position.

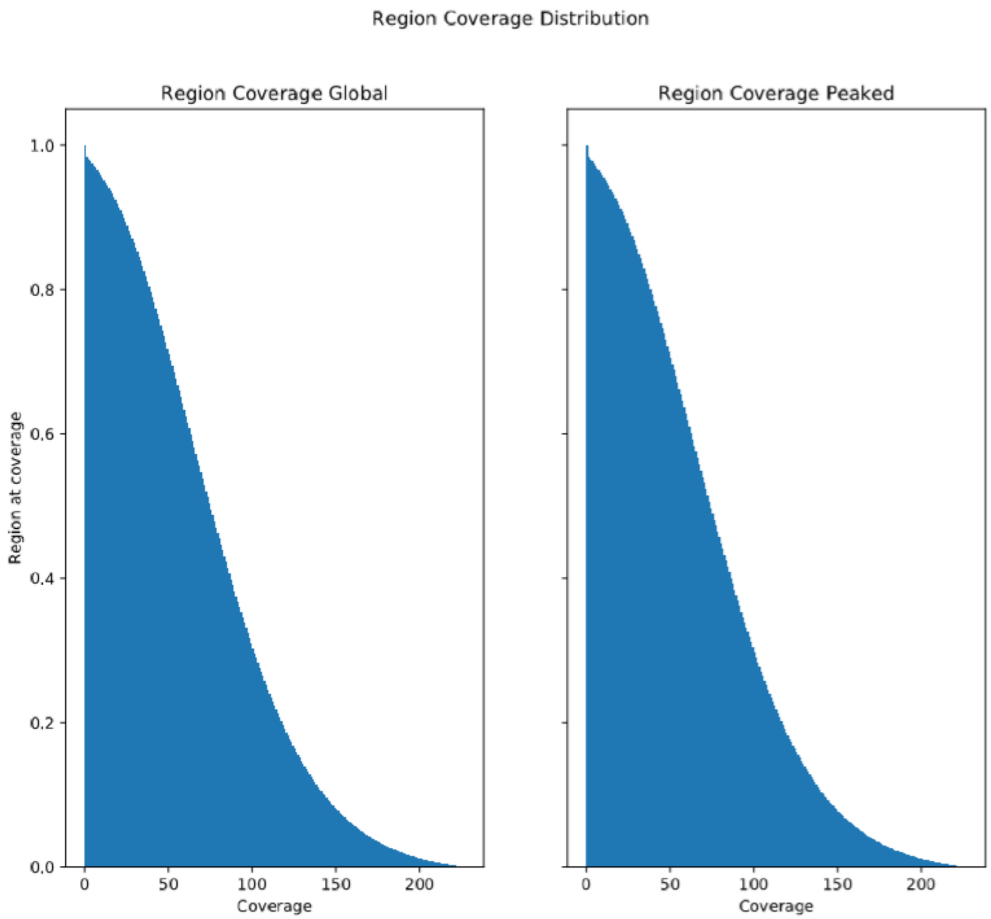


**Figure S7:** Genomic regions depth of coverage distribution report. Example of visual report of the mean depth of coverage distribution computed across all regions reported in the PaCBAM output file. Distribution is reported both for regions overall mean coverage and for regions fractions maximizing mean coverage.

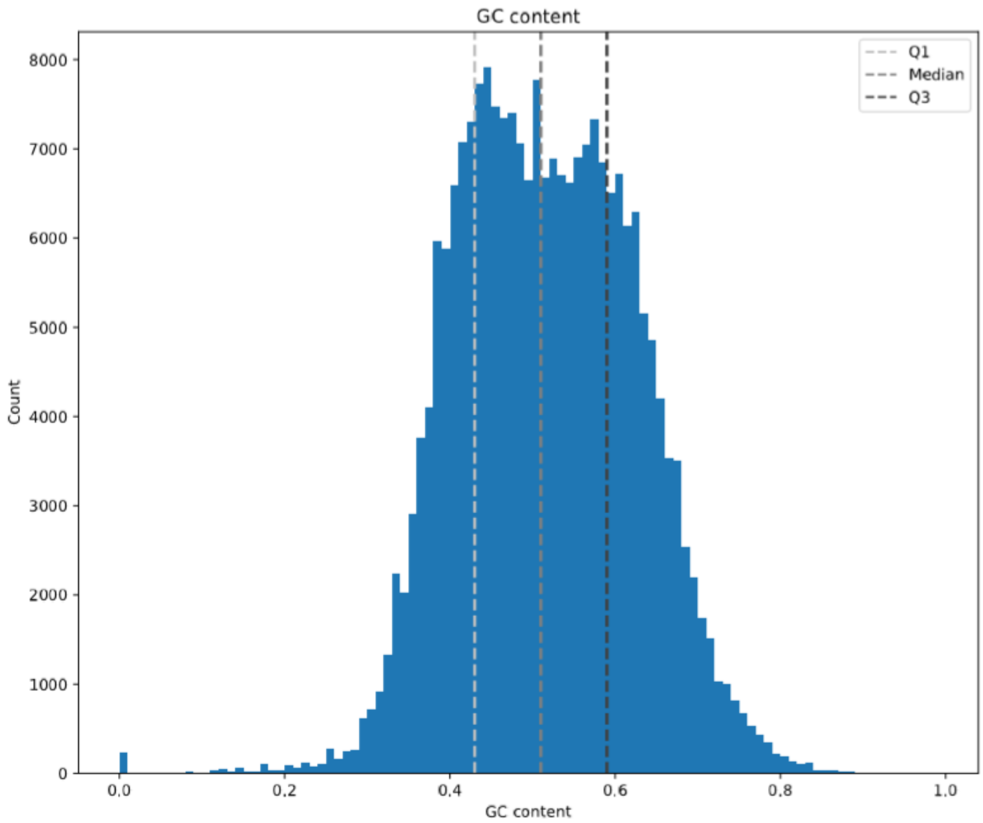


**Figure S8:** Genomic regions GC content distribution report. Example of visual report of the distribution of GC content computed across all regions reported in the *.rc PaCBAM output file.

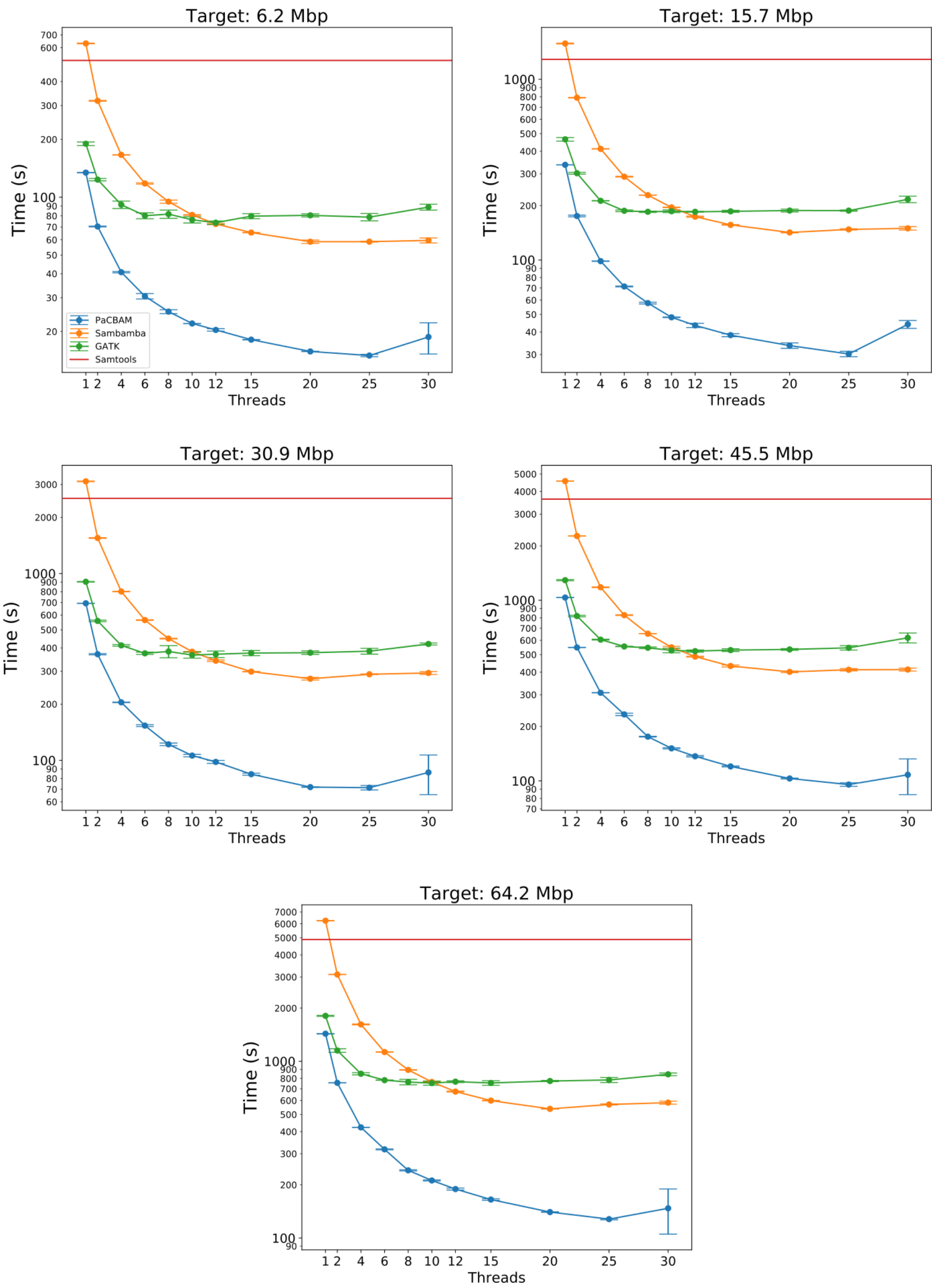


**Figure S9:** Run time comparison at 150X depth of coverage. Run time comparison among PaCBAM pileup and pileup module of SAMtools, GATK and Sambamba. Comparison is performed on BAM files at mean depth of coverage ~150X, at different target sizes and by increasing the number of threads.

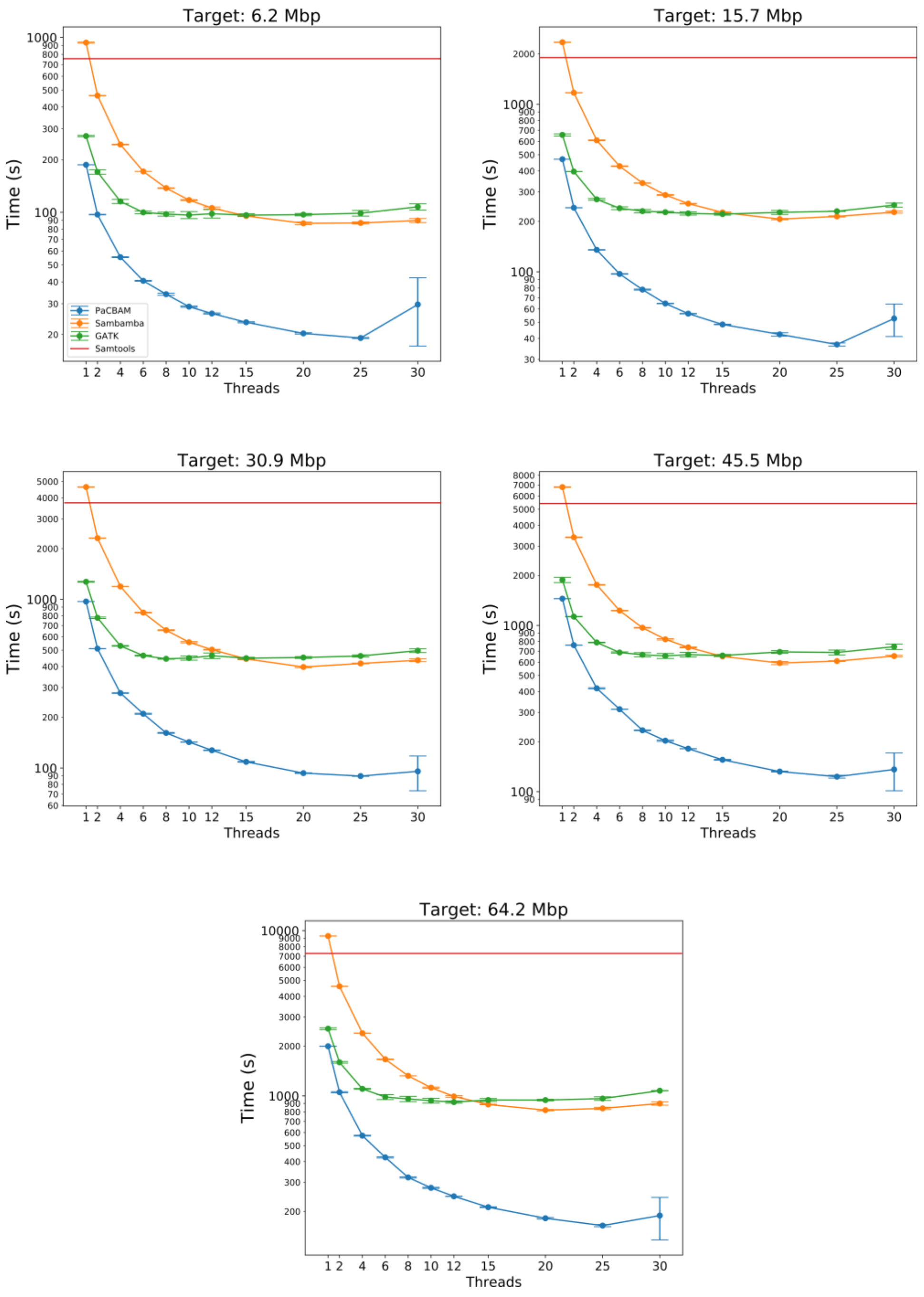


**Figure S10:** Run time comparison at 230X depth of coverage. Run time comparison among PaCBAM pileup and pileup module of SAMtools, GATK and Sambamba. Comparison is performed on BAM files at mean depth of coverage ~230X, at different target sizes and by increasing the number of threads.

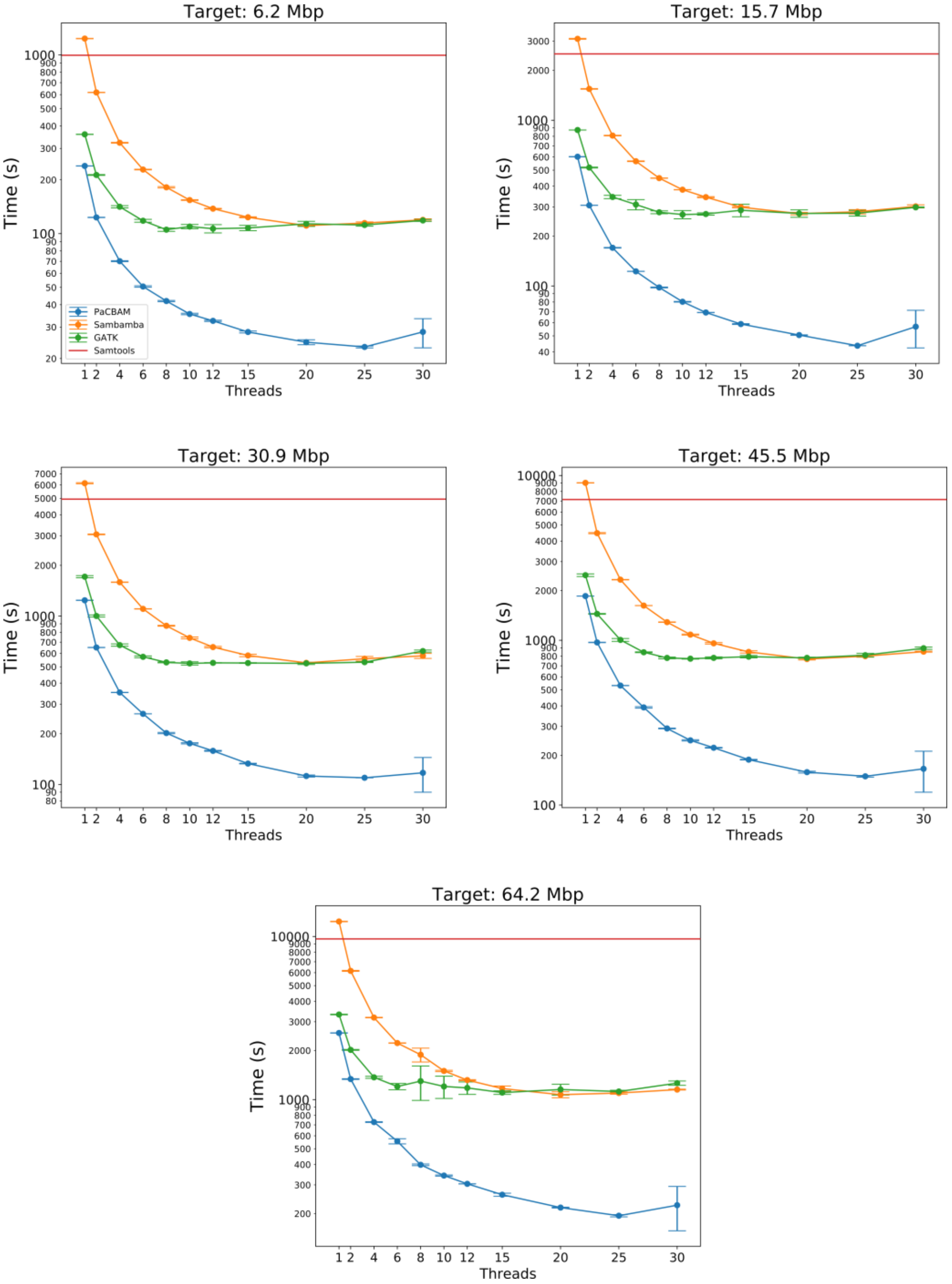


**Figure S11:** Run time comparison at 300X depth of coverage. Run time comparison among PaCBAM pileup and pileup module of SAMtools, GATK and Sambamba. Comparison is performed on BAM files at mean depth of coverage ~300X, at different target sizes and by increasing the number of threads.

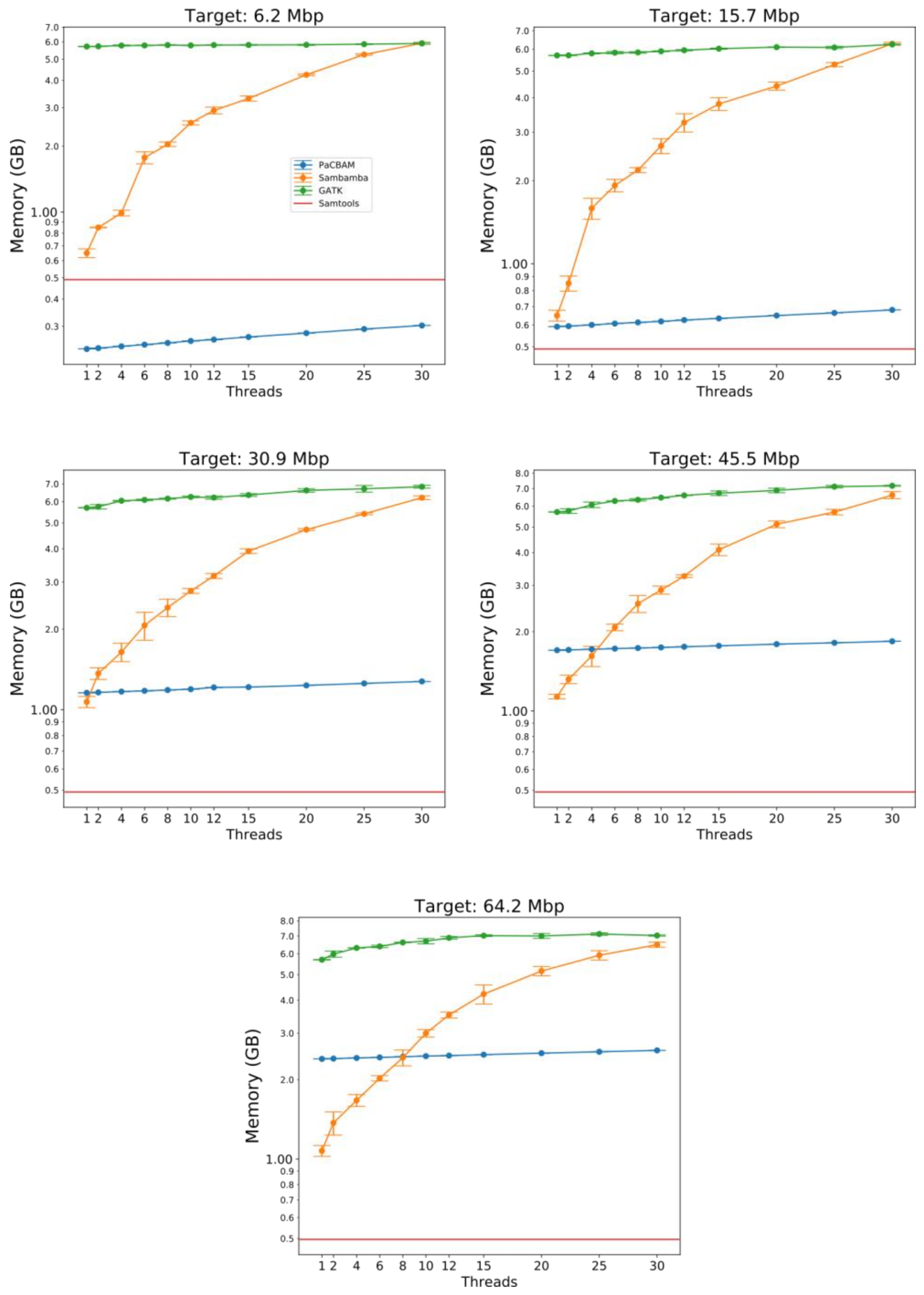


**Figure S12:** Memory usage comparison at 150X depth of coverage. Memory usage comparison among PaCBAM pileup and pileup module of SAMtools, GATK and Sambamba. Comparison is performed on BAM files at mean depth of coverage ~150X, at different target sizes and by increasing the number of threads.

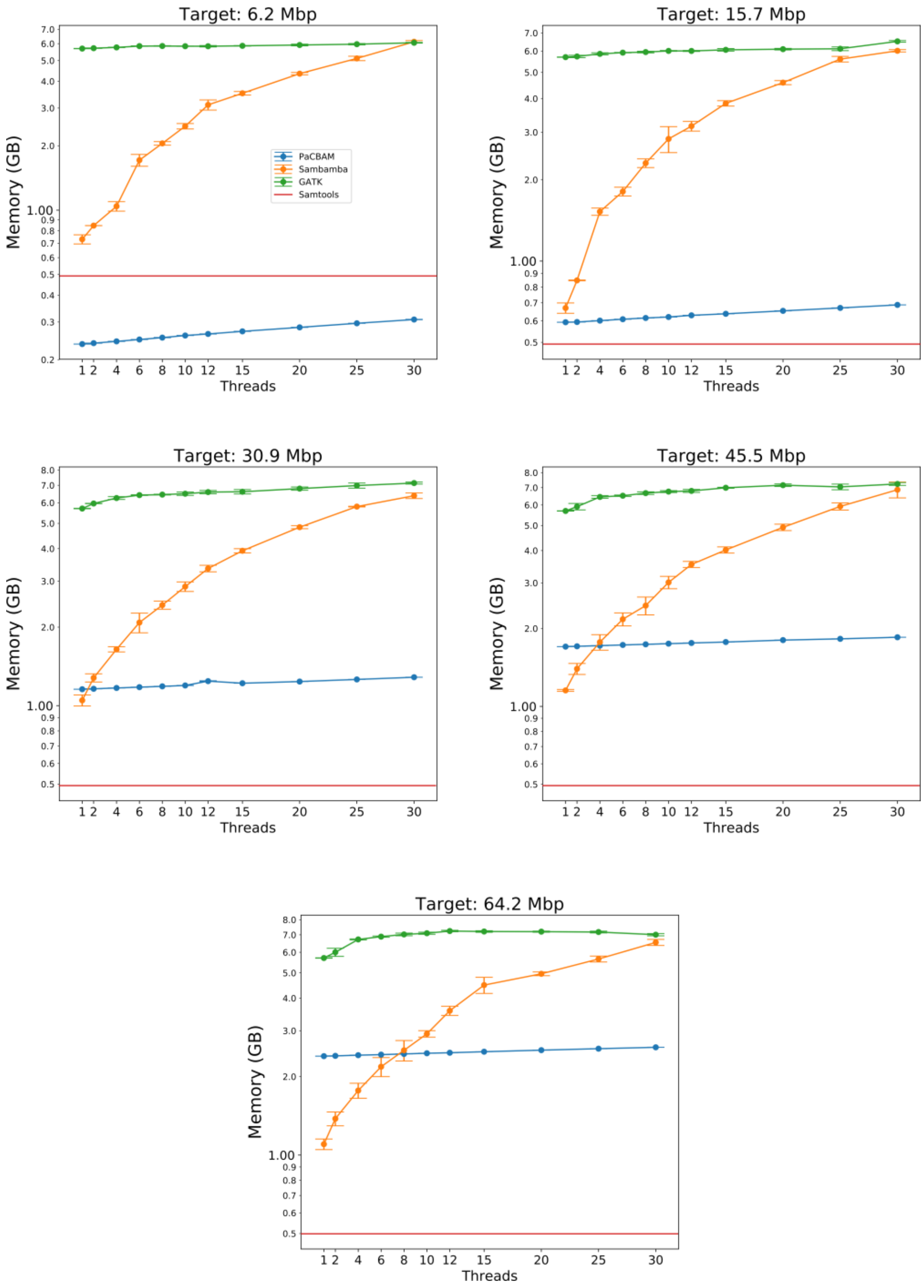


**Figure S13:** Memory usage comparison at 230X depth of coverage. Memory usage comparison among PaCBAM pileup and pileup module of SAMtools, GATK and Sambamba. Comparison is performed on BAM files at mean depth of coverage ~230X, at different target sizes and by increasing the number of threads.

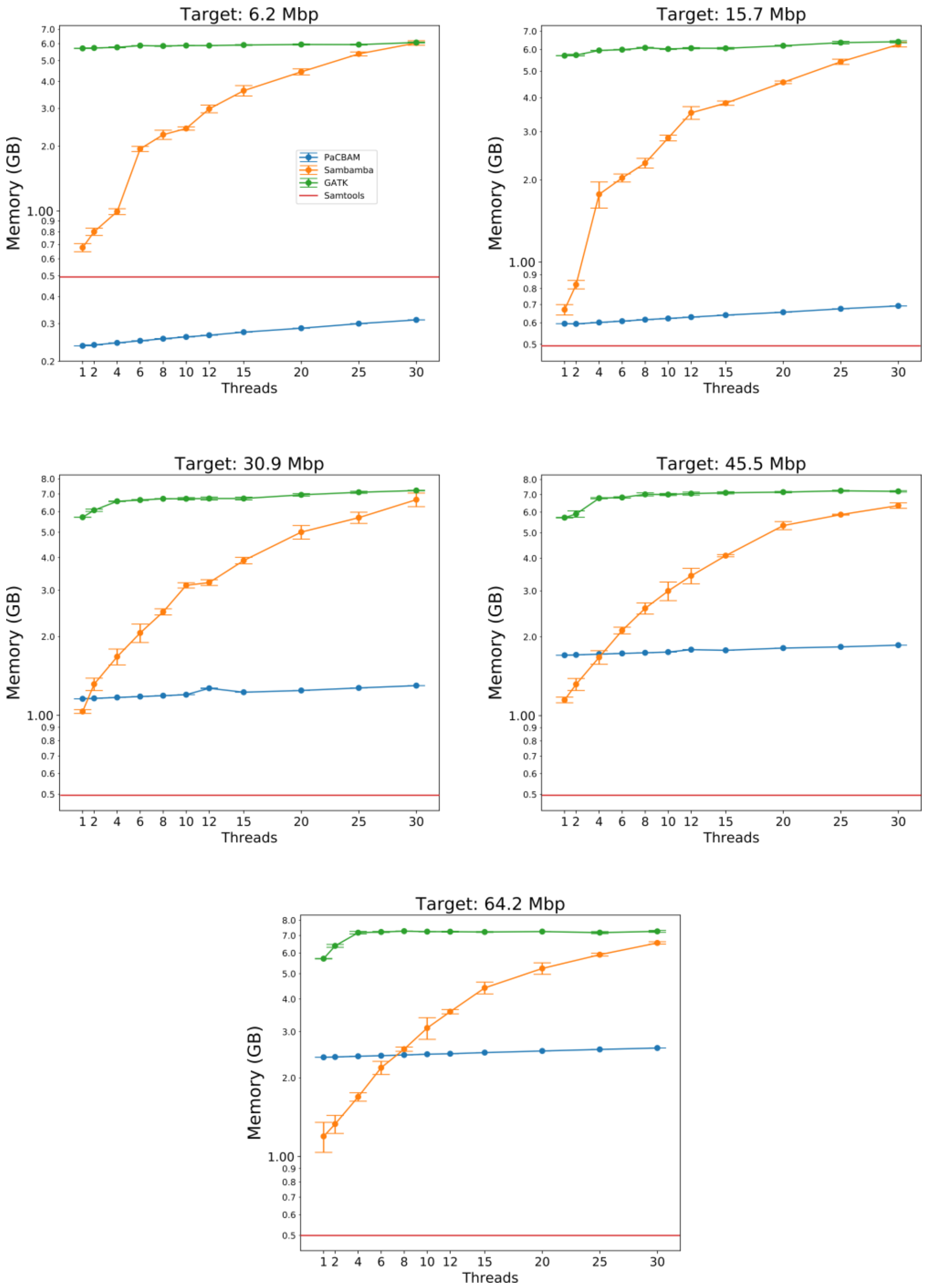


**Figure S14:** Memory usage comparison at 300X depth of coverage. Memory usage comparison among PaCBAM pileup and pileup module of SAMtools, GATK and Sambamba. Comparison is performed on BAM files at mean depth of coverage ~300X, at different target sizes and by increasing the number of threads.

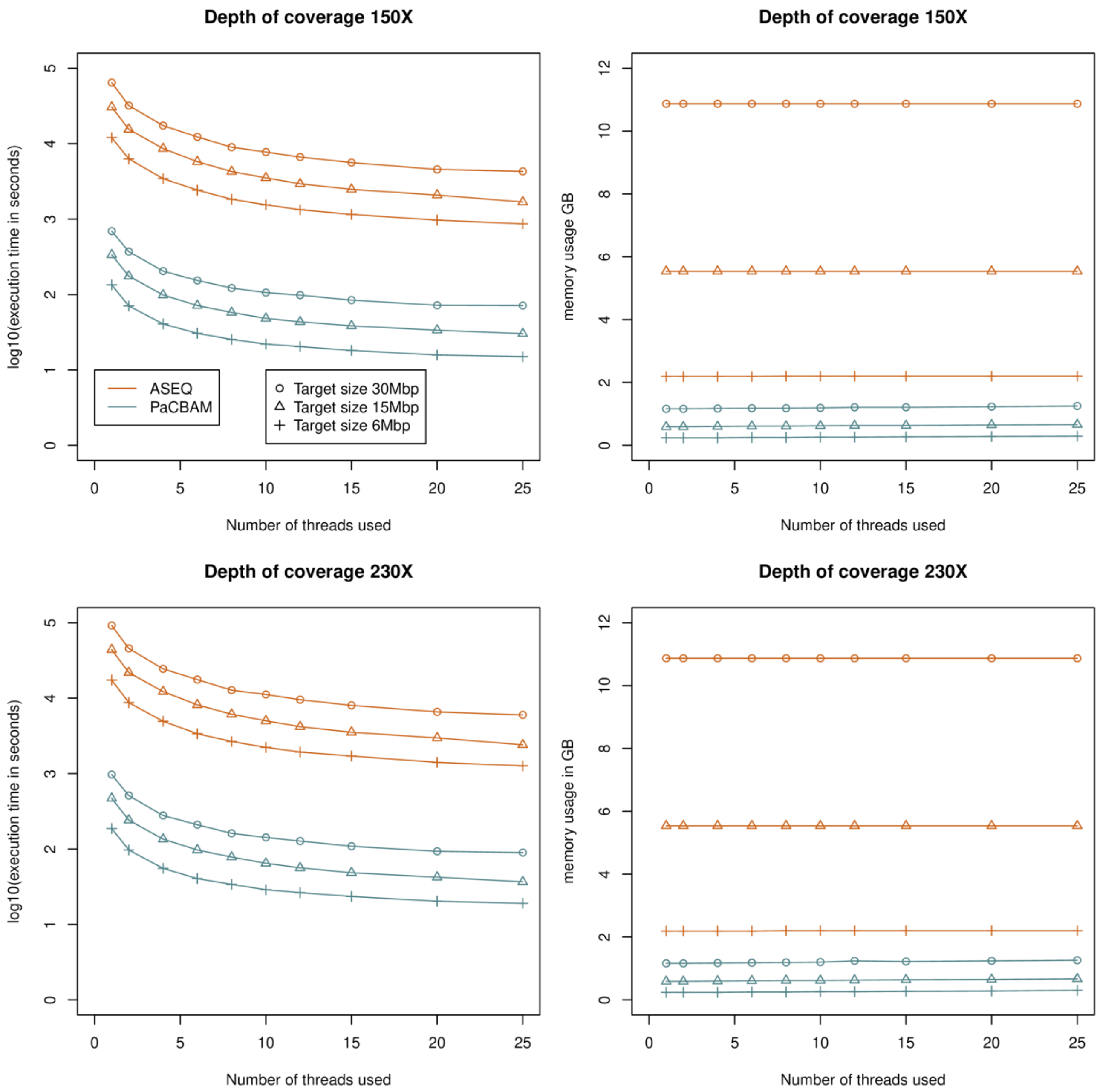


**Figure S15:** Memory usage comparison among PaCBAM pileup and pileup module of ASEQ. Comparison is performed on BAM files at mean depth of coverage ~150X and ~230X, at different target sizes and by increasing the number of threads.

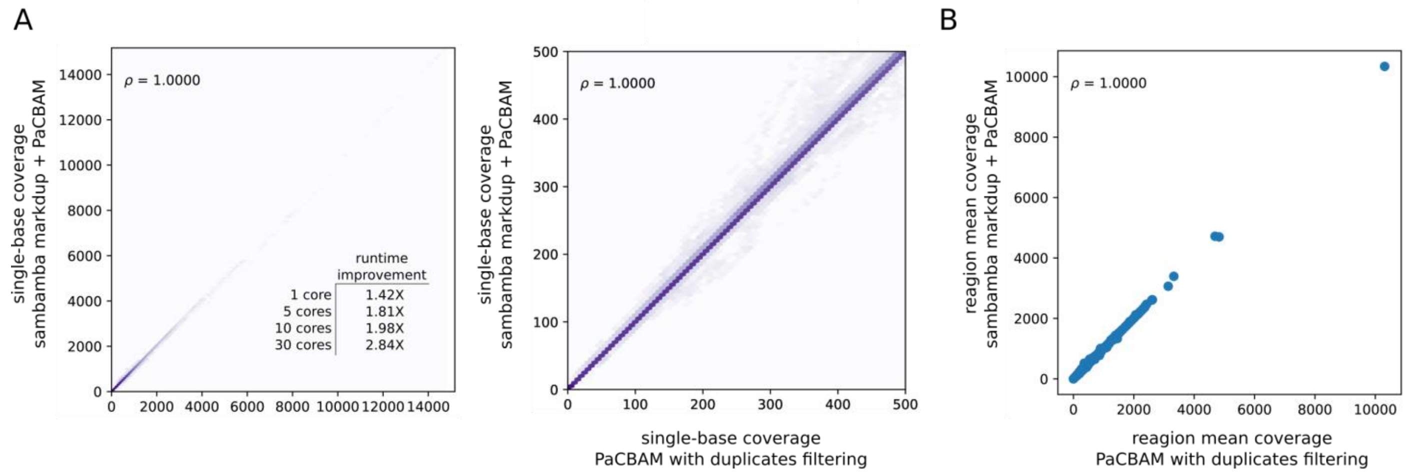


**Figure S16:** Comparison of PaCBAM duplicates filtering strategy to Sambamba markdup and Picard MarkDuplicates modules. A) Read duplicates filtering comparison between Sambamba markdup and PaCBAM (left) with zoom at smaller coverage interval (right). B) Regional mean depth of coverage of a BAM obtained by running either *sambamba markdup + PaCBAM pileup* or *PaCBAM pileup with duplicates filtering* option active. All results are highly concordant with correlation equal to 1.

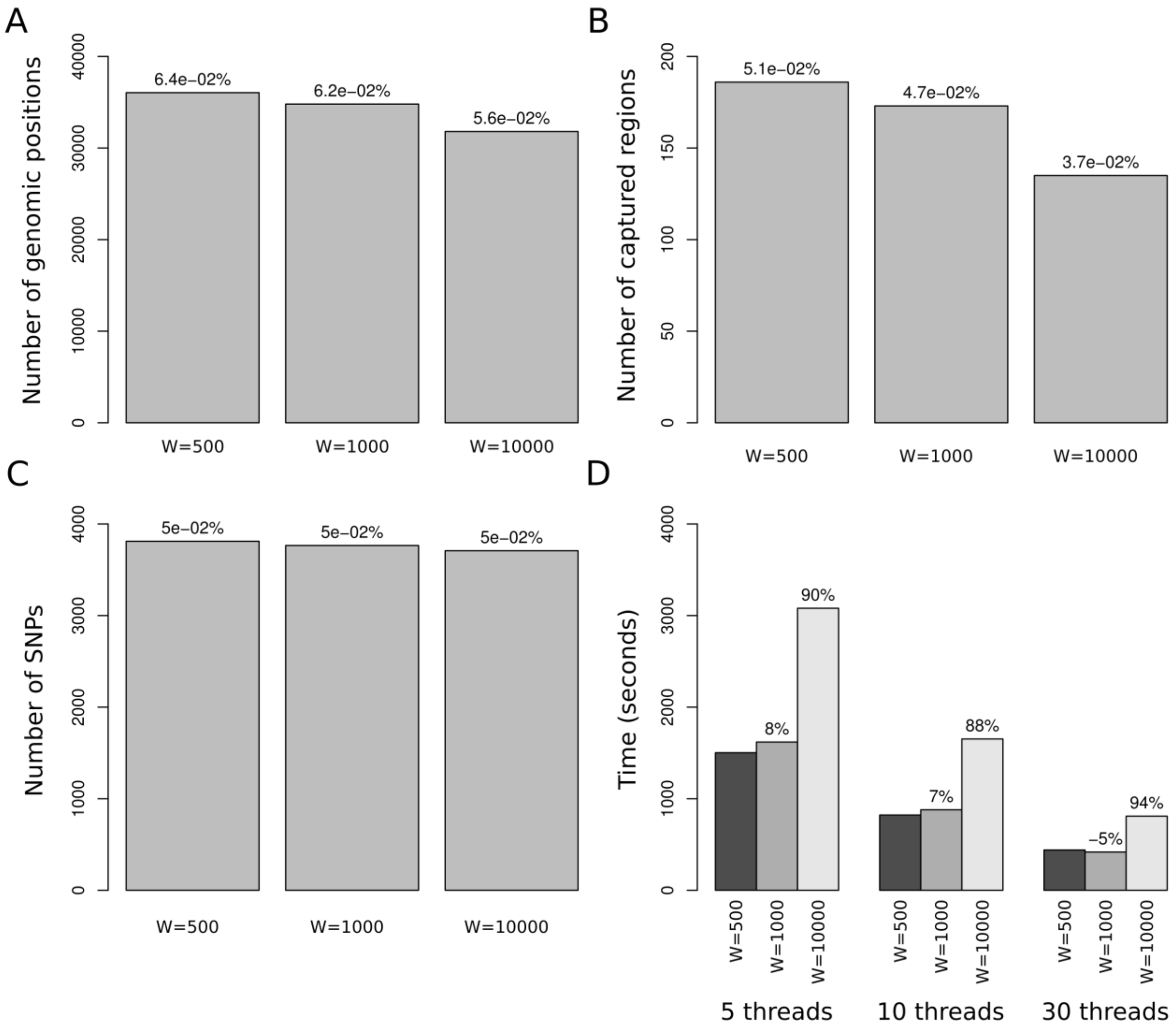


**Figure S17:** Performance of PaCBAM duplicated reads filtering. A) Number of positions that have a difference in coverage with respect to Picard results >=10 reads across different values for the *W* PaCBAM parameter; percentages on the bars are calculated with respect to the total number of genomic positions captured in the considered WES kit (N=56,379,320), excluding SNVs annotated in dbSNP v151. B) Number of regions that have a difference in coverage with respect to Picard results >=10 reads across different values for the *W* PaCBAM parameter; percentages on the bars are calculated with respect to the total number captured regions in the considered WES kit (N=368.146). C) Number of SNPs that have an allelic fraction difference with respect to Picard results >=1% across different *W* PaCBAM parameters; percentages on the bars are calculated with respect to the total number of SNVs annotated in dbSNP v151 and present in the WES kit (= 7,811,427). Results in panels A, B and C are computed comparing runs of PaCBAM with duplicated reads filtering and runs of PaCBAM on files output of Picard MarkDuplicates. D) Execution time of PaCBAM with duplicated reads filtering across different values for the *W* PaCBAM parameter and number of threads; percentages on the bar represent the time increase with respect to the previous bar value.

# Supplementary Tables

| Mean depth of coverage | Target size (bp) |
|---:|---:|
| 306.02 | 64,190,747 |
| 229.51 | 64,190,747 |
| 152.99 | 64,190,747 |
| 314.78 | 45,535,680 |
| 236.09 | 45,535,680 |
| 157.37 | 45,535,680 |
| 315.59 | 30,893,295 |
| 236.70 | 30,893,295 |
| 157.79 | 30,893,295 |
| 314.03 | 15,744,773 |
| 235.54 | 15,744,773 |
| 157.02 | 15,744,773 |
| 314.28 | 6,229,847 |
| 235.70 | 6,229,847 |
| 157.13 | 6,229,847 |

**Table S1:** Mean depth of coverage and target sizes of all BAM files used to test PaCBAM performance.

| Tools | Threads | Time (s) | Memory (MB) |
|---|---|---|---|
| DedupPaCBAM Duplicate Window 500 | 1 | 6557.00 | 3823.49 |
| DedupPaCBAM Duplicate Window 500 | 5 | 1502.66 | 3878.53 |
| DedupPaCBAM Duplicate Window 500 | 10 | 821.73 | 3937.54 |
| DedupPaCBAM Duplicate Window 500 | 30 | 440.45 | 4182.63 |
| DedupPaCBAM Duplicate Window 1000 | 1 | 7082.33 | 3823.46 |
| DedupPaCBAM Duplicate Window 1000 | 5 | 1618.61 | 3887.65 |
| DedupPaCBAM Duplicate Window 1000 | 10 | 879.37 | 3959.21 |
| DedupPaCBAM Duplicate Window 1000 | 30 | 417.96 | 4236.96 |
| DedupPaCBAM Duplicate Window 10000 | 1 | 13105.67 | 3853.50 |
| DedupPaCBAM Duplicate Window 10000 | 5 | 3080.07 | 3941.18 |
| DedupPaCBAM Duplicate Window 10000 | 10 | 1652.61 | 4052.34 |
| DedupPaCBAM Duplicate Window 10000 | 30 | 809.34 | 4569.39 |
| Sambamba markdup + PaCBAM pileup | 1 | 9308.40 | 3802.29 |
| Sambamba markdup + PaCBAM Pileup | 5 | 2712.57 | 5290.98 |
| Sambamba markdup + PaCBAM Pileup | 10 | 1626.27 | 7441.42 |
| Sambamba markdup + PaCBAM Pileup | 30 | 1251.32 | 18473.83 |
| Picard markdup + PaCBAM Pileup | 1 | 12422.76 | 28994.42 |
| Picard markdup + PaCBAM Pileup | 5 | 10764.17 | 28994.42 |
| Picard markdup + PaCBAM Pileup | 10 | 10541.08 | 28994.42 |
| Picard markdup + PaCBAM Pileup | 30 | 10396.25 | 28994.42 |

**Table S2:** Time and memory usage of duplicates filtering performance analyses. When combining MarkDuplicates and PaCBAM the memory usage is the peak memory usage of the entire pipeline.

| **Tool** | **Version** |
| --- | --- |
| Sambamba | 0.6.8-pre1 compiled with LDC 1.8.0 and LLVM 5.0.1 |
| GATK | 3.8-0-ge9d806836 |
| SAMtools | 1.7 |
| Picard MarkDuplicates | 2.17.4 |

**Table S3:** Versions of the tools used in performance evaluation analysis.
